# Incidence of adverse events in iron‐deficient pregnant women and surgical patients undergoing intravenous iron treatment with ferric isomaltose or ferric carboxymaltose: A systematic review

**DOI:** 10.1111/trf.70105

**Published:** 2026-02-02

**Authors:** Dominik Heger, Johannes Volkmann, Stephanie Weibel, Johanna Stoevesandt, Veronika Walzer, Peter Kranke, Patrick Meybohm, Stephanie Stangl

**Affiliations:** ^1^ Department of Anaesthesiology, Intensive Care, Emergency and Pain Medicine University Hospital Würzburg Würzburg Germany; ^2^ Department of Dermatology, Venereology, and Allergology University Hospital Würzburg Würzburg Germany

**Keywords:** adverse events, intravenous iron, pregnancy, surgery

**Abbreviations**
AE: adverse event
CASP: Critical Appraisal Skills Programme
FCM: ferric carboxymaltose
FDM: ferric (der‐)isomaltose
HP: hypophosphatemia
HSR: hypersensitivity reaction
IDA: iron deficiency anemia
IVI: intravenous iron
RCT: randomized controlled trial
TSAT: transferrin saturation

## 1 INTRODUCTION

Iron deficiency anemia (IDA) remains the most common form of anemia worldwide, with pregnant women particularly at risk due to increased iron demand.^1^, ^2^, ^3^, ^4^ Despite increased awareness and a slight decline since the early 2000s, approximately 37% of pregnant women worldwide still suffer from anemia, which is associated with an increased risk of peripartum hemorrhage and contributes to around 19% of maternal deaths.^5^, ^6^ This has prompted global health efforts, including the Millennium Development Goals, to improve maternal outcomes.^7^ Surgical patients with iron deficiency with or without anemia (ID(A)) are also vulnerable, especially in the perioperative setting. Intravenous iron (IVI) is an established element of Patient Blood Management, offering advantages over oral iron, which is often poorly tolerated due to gastrointestinal adverse events (AEs).^8^, ^9^, ^10^, ^11^, ^12^, ^13^, ^14^, ^15^, ^16^, ^17^ Newer third‐generation IVI formulations like ferric carboxymaltose (FCM) and ferric (der‐)isomaltose (FDM) allow for high‐dose administration with potentially lower rates of AEs.^18^, ^19^, ^20^, ^21^, ^22^ Still, specific AEs such as hypophosphatemia (HP), hypersensitivity reactions (HSRs), and anaphylaxis remain concerns.^23^, ^24^ HP, frequently subclinical, is linked to altered iFGF23 metabolism and can cause symptoms overlapping with ID, including fatigue or osteomalacia.^25^, ^26^, ^27^, ^28^, ^29^, ^30^, ^31^, ^32^, ^33^ Distinguishing HSRs from anaphylaxis is challenging due to overlapping symptoms and inconsistent terminology.^34^, ^35^, ^36^ The umbrella term hypersensitivity is generally used to encompass allergic reactions of the immediate type (IgE‐mediated, anaphylaxis) as well as the delayed type (e.g., exanthema). Anaphylaxis is an acute, systemic reaction triggered by the rapid release of mediators from mast cells and basophils, leading to potentially life‐threatening symptoms such as airway obstruction, circulatory collapse, and cutaneous manifestations. It can have an allergic cause (IgE‐mediated, immediate type hypersensitivity) or be non‐allergic in origin. Complement activation‐related pseudoallergy (CARPA) has been proposed as a possible mechanism of non‐allergic anaphylaxis, though its relevance in pregnancy—where complement activity is elevated—remains unclear.^35^, ^36^, ^37^, ^38^ Existing reviews did not require lab‐confirmed ID and did not report on HP, HSRs, or anaphylaxis specifically.^21^, ^39^ As IVI use is increasing, assessing these AEs in patients with verified ID is essential to avoid unnecessary risks. Standard markers like ferritin and transferrin saturation (TSAT) are critical for diagnosis.^40^ This systematic review aims to evaluate the incidence and severity of HP, HSRs, and anaphylactic reactions following IVI treatment with FCM or FDM in pregnant and surgical patients with laboratory‐confirmed ID. In addition, the study investigates the reporting quality and classification of these AEs across eligible trials.

## 2 METHODS

We prospectively registered the study protocol in PROSPERO with identifier CRD42023428997.^41^

### 2.1 Search strategy and study selection

We conducted a systematic search of MEDLINE (via Ovid), CENTRAL (via Cochrane Library), ClinicalTrials.gov, the WHO International Clinical Trials Registry Platform (ICTRP), and from the website of regulatory bodies (e.g., European Medicine Agency (EMA)) Clinical Trials Register (EUCTR) up to May 23, 2023. Detailed search strategies are provided in Table S1.

Search results were imported into EndNote, and duplicates were removed before transferring the records into Covidence, where additional duplicate entries were identified and removed. Two independent reviewers screened all references at the title and abstract level against predefined exclusion criteria. Disagreements were resolved through consultation with a third reviewer or group discussion.

Full‐text articles of potentially eligible studies were then retrieved and assessed independently by two reviewers. As recommended by the *Cochrane Handbook for Systematic Reviews of Interventions*, we sought additional information for included studies, such as clinical study reports or data from EUCTR, to ensure comprehensive analysis.^42^

### 2.2 Eligibility criteria

We included studies published after 2000 that reported the incidence of AEs associated with IVI treatment for ID(A) in pregnant women (aged ≥18 years) or non‐pregnant surgical patients (aged ≥18 years). Eligible studies included randomized controlled trials (RCTs) and cohort studies that compared FCM or FDM with oral iron preparations, placebo, standard care (e.g., red blood cell transfusion), or each other in head‐to‐head comparisons.

Inclusion required laboratory‐confirmed ID based on hematological parameters, such as ferritin levels or TSAT. Studies published in English or German were eligible for inclusion.

Exclusion criteria encompassed studies involving health conditions associated with secondary ID(A), such as sickle cell disease, malaria, or patients requiring hemodialysis.

### 2.3 Data extraction

Data extraction was performed using a predefined, piloted Excel sheet. Each extraction was independently verified by a second reviewer, with discrepancies resolved through discussion or consultation with a third reviewer, as in the screening process. Extracted data are presented in Table S2.

Initial plans to extract AEs data based solely on explicit mentions in publications proved inadequate for addressing our primary research question. An allergist was consulted to define the terms “anaphylaxis” and “HSR” based on their possible clinical symptoms.

Given the heterogeneity and lack of standardized reporting of AEs, which would have led to an underestimation of the incidence of AEs, the review authors developed an alternative approach. This involved comparing AEs explicitly labeled as HP, HSRs, or anaphylaxis with the symptom complexes reported in the studies (e.g., urticaria/angioedema/itching, and/or bronchospasm, and/or hypotension/loss of consciousness for anaphylaxis). These symptom complexes were also compared against a comprehensive symptom spectrum derived from multiple sources, including:

- Manufacturer information for Ferinject® and MonoFer®
- Clinical data cited by manufacturers regarding IV iron and HSRs^43^
- Reviews on HSRs to IVI^35^
- Current allergology literature^44^, ^45^, ^46^

The resulting standardized symptom list is provided in Table S4. Using this framework, we extracted the number of symptom events reported in each study. Where possible, we supplemented this with additional information obtained outside the primary publications.

Additional methodological details regarding AE assessment were also extracted to identify differences in outcome measurement and reporting. Specific criteria included the use of standardized taxonomies (e.g., MedDRA [Medical Dictionary for Regulatory Activities]) and potential limitations such as thresholding or selective reporting (e.g., focusing only on the intervention group). We also evaluated whether the methods sections of included studies adequately described protocols for assessing unwanted events, including harms and AEs.

This approach aimed to highlight discrepancies in AE reporting, providing a clearer picture of the challenges in interpreting outcomes related to HP, HSRs, and anaphylaxis in the included studies.

### 2.4 Quality assessment

The quality of included studies was evaluated using the Critical Appraisal Skills Programme (CASP) checklists for RCTs and cohort studies.^47^ Two independent reviewers conducted the assessments, with disagreements resolved through discussion or, when necessary, consultation with a third reviewer.

## 3 RESULTS

### 3.1 Study selection

Our search identified 6731 records across the specified databases and registers. After the automated removal of 734 duplicates using EndNote and Covidence, an additional four duplicates were manually removed, resulting in 5993 records for title and abstract screening. Two independent reviewers assessed these records for eligibility, excluding 5625 records that did not meet the criteria.

**FIGURE 1:**
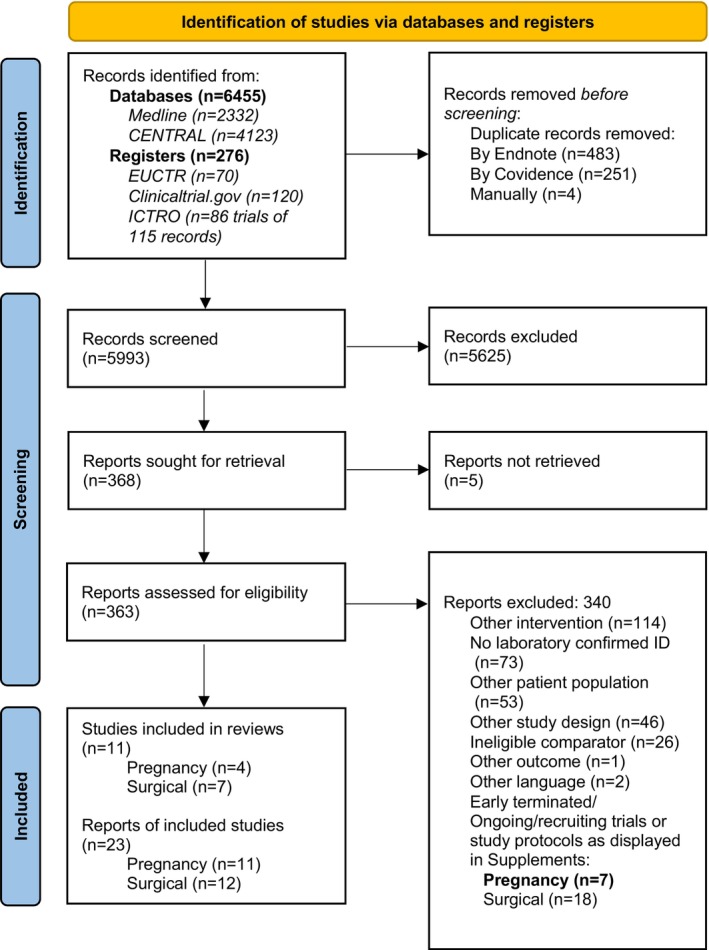
PRISMA flowchart of study selection.

This left 368 reports, from which 363 full‐text articles were retrieved and assessed for eligibility. Reasons for further exclusion in process are detailed in the PRISMA flow diagram (Figure 1).

Ultimately, 11 studies were included in this systematic review:

- Prenatal ID therapy in pregnant women: 4 RCTs
- Preoperative ID therapy in non‐pregnant surgical patients: 7 studies (5 RCTs and 2 cohort studies)

### 3.2 Study characteristics

Among the included studies, three out of four studies in pregnant women used FCM,^48^, ^49^, ^50^ while Hansen et al. used FDM.^51^ For surgical patients, six studies used FCM,^52^, ^53^, ^54^, ^55^, ^56^, ^57^ and one study used FDM.^58^

The multi‐center study by Breymann et al. was conducted across six countries (South Korea, Russia, Australia, Singapore, Sweden, and Switzerland), while four studies were conducted in Europe (Denmark, Netherlands, Italy, and Spain), two in India, three in Asian countries (Hong Kong, Korea, and Singapore), and one in Australia.

### 3.3 Diagnosis of ID

ID was diagnosed using various criteria across studies. Four studies used serum ferritin alone as a predefined threshold, while five studies combined ferritin with TSAT. One study used TSAT as the sole diagnostic criterion.^55^ Another study did not specify a hematological parameter for ID but excluded anemia not related to ID(A), allowing for its inclusion in this systematic review.^49^

The characteristics of the included studies are summarized in Table 1, with details of excluded ongoing studies provided in Table S3.

**TABLE 1:** Study characteristics of included studies.

Study (year), registration number	Country	Study type	Follow‐up duration	Diagnostic criteria anemia, ID	Population	Age (years), mean (±SD)	Intervention intravenous iron preparation	Control group
Studies of prenatal ID therapy in pregnant women with intravenous iron formulations								
Breymann et al. (2017)^48^ NCT01131624	South Korea, Russia, Australia, Singapore, Sweden, Switzerland	RCT	Up to 12 weeks after baseline assessment (or delivery) whatever came first	[Hb] ≤11 g/dL,^a^ serum ferritin levels ≤ 20 ng/mL	Pregnant women with IDA aged 18 years or older within their 2nd or 3rd trimester Singleton only	FCM 31.4 (±5.77) FS 30.9 (±5.39)	FCM, single or multiple administration, *n* = 123 Cumulative dosage: 1000 mg for [Hb] >9 g/dL; 1500 mg for [Hb] ≤ 9 g/dL Number of administrations depending on pre‐pregnancy body weight	FS, p.o. *n* = 124, 100 mg iron (twice daily)
Chawla et al. (2022)^49^ CTRI/2017/06/008884	India	RCT	Up to 6 weeks after baseline assessment	[Hb] <11 g/dL, no cut‐off declared^b^	Pregnant women with IDA aged 18 years or older between 18 and 24 POG (weeks)	FCM 26.43 (±3.6) FS 26.26 (±3.46)	FCM, single administration, *n* = 181, 1000 mg	FS, p.o. *n* = 181 60 mg iron (twice daily)
Hansen et al. (2023)^51^ NCT03188445	Denmark	RCT	Up to 18 weeks after baseline assessment	[Hb] <11 g/dL, serum ferritin levels ≤30 ng/mL	Pregnant women with ID aged 18 years or older within their 2nd trimester Singleton only	FDM 30.7 (±4.5) FF‐AC 31.3 (±4.6)	FDM, single administration, *n* = 100, 1000 mg^c^	FF‐AC, p.o. *n* = 101,100/60 mg iron/ascorbic acid once a day
Khalafallah et al. (2018)^50^ ACTRN 12613000853741	India	RCT	Up to 4 weeks after baseline assessment and 24 h prior to delivery	[Hb] <11 g/dL, serum ferritin levels ≤100 ng/mL or TSAT ≤ 20%	Pregnant women with IDA aged 18 years or older within their 2nd or 3rd trimester	FCM 28.7 (±5.99) FS 28.5 (±6.3)	FCM, single administration, *n* = 83 1000 mg	FS, p.o. *n* = 81,105 mg iron once a day
Studies of preoperative ID therapy in surgical patients with intravenous iron formulations								
Thin et al. (2021)^52^ NCT03295851	Singapore	RCT	Up to 12 weeks after baseline assessment	[Hb]_male_ <13 g/dL [Hb]_female_ <12 g/dL, serum ferritin levels <100 μg/L or 100–300 μg/L and TSAT ≤20%	21 years or older with IDA, undergoing elective major abdominal surgery	FCM 59.2 (±12.4) FF 55.2 (±23.3)	FCM, single administration, *n* = 15 1000 mg	FF, p.o., *n* = 15, 200 mg (twice daily)
Froessler et al. (2016)^11^ ACTRN12611000387921	Australia	RCT	Up to 4 weeks after baseline assessment	[Hb]_male_ <13 g/dL [Hb]_female_ <12 g/dL Serum ferritin levels <300 μg/L TSAT <25%	18 years or older with IDA undergoing major abdominal surgery	FCM 64 (±15) SoC 68 (±15)	FCM, single administration, *n* = 40, 1000 mg	SoC, *n* = 32
Kim et al. (2023)^54^ NCT04898569	Korea	RCT	Up to 12 weeks after baseline assessment	[Hb]_male_ <13 g/dL [Hb]_female_ < 12 g/dL Serum ferritin levels <300 μg/L TSAT ≤25%	19 years or older with IDA undergoing elective OPCAB or MIDCAB	FCM 70.6 (±8.3); Placebo 72.2 (±9.2)	FCM, single administration *n* = 431,000 mg	Placebo *n* = 43
Talboom et al. (2023)^55^ NCT02243735	Netherlands	RCT	Up to 6 months after baseline assessment	[Hb]_male_ <13 g/dL [Hb]_female_ <12 g/dL TSAT ≤20%	19 years or older with IDA undergoing curative resection for M0 stage colorectal cancer	FCM 72 (IQR 63–79) FF 70 (IQR 61–81)	FCM, single or multiple administration, *n* = 96 Cumulative dosage: Between 2000 mg (for [Hb] ≤10 g/dL and body weight over 70 kg) and 1000 mg (for [Hb] >10 g/dL and body weight 35–70 kg)	FF, p.o. *n* = 106, 200 mg once a day until day of surgery
Fung et al. (2022)^58^ NCT03565354	Hong Kong	RCT	From baseline assessment until hospital discharge	[Hb] <13 g/dL Serum ferritin <30 μg/L or 30–100 μg/L TSAT ≤20%	18 years or older with IDA undergoing elective colorectal cancer surgery	FDM 68.4 (±6.8) SoC 69.8 (±12.6)	FDM, single administration, *n* = 20, 1000 mg	SoC, *n* = 20
D'Amato et al. (2020)^56^	Italy	Cohort	Up to 4 weeks after baseline assessment	NA Serum ferritin levels <100 μg/L (and displaying normal CRP values)	Patients, aged 35–80 years, with ID undergoing hip and knee arthroplasty	FCM 67.6 (±9.5) SoC 66.0 (±10.9)	FCM, single administration, *n* = 83, 1000 mg	SoC, *n* = 62
Calleja et al. (2016)^57^	Spain	Cohort	Up to 1 month after baseline assessment	[Hb]_male_ <13 g/dL [Hb]_female_ <12 g/dL Serum ferritin levels <30 ng/mL, TSAT ≤ 20%	18 years or older with IDA undergoing elective curative surgery for adenocarcinoma of the colon	FCM 72.9 (±11.1) SoC 70.8 (±10.3)	FCM, *n* = 111 Median total dose 1000 mg (1275 ± 430.1)	Oral iron, *n* = 155 (different doses and preparations)

Abbreviations: ACTRN, The Australian and New Zealand Clinical Trial Registry; CRP, C‐reactive protein; CTCAE, Common Terminology Criteria for Adverse Events; CTRI, clinical Trials Registry‐India; FCM, ferric carboxymaltose (Ferinject®); FDM, ferric derisomaltose (MonoFer®); FF‐AC, ferrous fumarate with ascorbic acid; FS, ferrous sulfate; Hb, hemoglobin (level); ID, iron deficiency; ID(A), iron deficiency (anemia); IQR, interquartile range; MedDRA, Medical Dictionary for Regulatory Activities; MIDCAB, minimally invasive direct coronary artery bypass; NA, not applicable; NCT, ClinicalTrials.gov registry number; OPCAB, off‐pump coronary artery bypass grafting; p.o., per os; POG, period of gestation; RCT, randomized controlled trial; SD, standard deviation; SoC, standard of care; TSAT, saturation of transferrin.

^a^ For gestation weeks 16–26 [Hb] ≤10.4 g/dL.

^b^ Anemia other than ID defined as exclusion criteria | in correspondence with author.

^c^ If pre‐pregnancy body weight <50 kg, the dose was 20 mg/kg pre‐pregnancy body weight | two women from the intervention group received one additional dose and 15 women from the control group also received one dose FDM at week 6 or 12.

### 3.4 AEs

None of the included studies explicitly reported HP as an AE, nor did they provide phosphate level measurements. Breymann et al.^48^ recorded 10 cases of reduced phosphate levels among 123 FCM‐treated pregnant women, but these were deemed clinically irrelevant. Attempts to infer HP occurrences based on reported symptoms associated with HP proved impractical. HP is asymptomatic in most cases, and when symptoms do occur, they are diverse, often nonspecific, and may overlap with symptoms of ID(A).^26^, ^27^, ^28^, ^29^, ^30^, ^31^ Consequently, no aggregation of symptoms potentially indicative of HP was conducted.

At first glance no anaphylactic reactions seemed to be reported among the 775 patients treated with FCM or the 120 patients treated with FDM across the included studies. But the reason for this was that three anaphylactic reactions were just not clearly stated as “anaphylaxis” by the study authors. Therefore, we decided that the following events should be labeled as anaphylactic reaction: *n* = 1 bronchospasm^48^ as “probable anaphylactic reaction”; *n* = 2 “bronchospasm, pruritus, and flushing”^51^ as “possible anaphylactic reaction.” Table 2 provides an overview of all AEs reported in the included studies.

**TABLE 2:** Overview of reported adverse events by all included studies.

AE	Total number of reported events	Number of studies reporting these AEs
HSRs | anaphylactic reaction	3	2
HP	0	0

Abbreviations: AE, adverse event; HP, hypophosphatemia; HSR, hypersensitivity reaction.

### 3.5 HSRs

HSRs were reported in two studies. Breymann et al.^48^ documented one case of bronchospasm classified as an HSR related to FCM administration. Hansen et al.^51^ reported two HSR cases with symptoms including bronchospasm, pruritus, and flushing following FDM administration.

Table 3 provides a detailed summary of the reported HSRs, listing the symptoms that led authors to classify the events as HSRs. Notably, Breymann et al.^48^ described a single symptom (bronchospasm) for their reported HSR, while Hansen et al.^51^ identified three symptoms (bronchospasm, flushing, and pruritus). The third column of Table 3 lists all reported symptoms potentially associated with HSRs, as derived from our methodology. A comprehensive list of symptoms linked to HSRs can be found in Table S4.

**TABLE 3:** Summary of incident hypersensitivity cases reported.

Author (year)	Number of reported HSRs	Number of symptoms declared HSRs by author	Number of all symptoms possibly related to HSRs^a^	Interruption or further treatment due to AEs	Additional sources used^b^	Established outcome taxonomy
Breymann et al. (2016)^48^	HSRs: *n*_FCM_ = 1/123	HSRs: *n*_FCM_ = 1/123	HSRs: *n*_FCM_ = 46/123	Yes (*n* = 1 due to HSR)	EudraCT protocol	MedDRA (version 16.1)
Hansen et al. (2022)^51^	HSRs: *n*_FDM_ = 2/100	HSRs: *n*_FDM_ = 6/100	HSRs: *n*_FDM_ = 76/100	Yes (*n* = 2 due to HSRs)	EudraCT protocol	MedDRA (version 20.0)
Chawla et al. (2022)^49^	HSRs: *n*_FCM_ = 0/181	HSRs: *n*_FCM_ = 0/181	HSRs: *n*_FCM_ = 10/181	No	NA	No
Khalafallah et al. (2018)^50^	HSRs: *n*_FCM_ = 0/83	HSRs: *n*_FCM_ = 0/83	HSRs: *n*_FCM_ = 6.3%	No	NA	No
Thin et al. (2021)^52^	HSRs: *n*_FCM_ = 0/13	HSRs: *n*_FCM_ = 0/13	HSRs: *n*_FCM_ = 0/13	No	NA	NA
Froessler et al. (2016)^53^	HSRs: *n*_FCM_ = 0/40	HSRs: *n*_FCM_ = 0/40	HSRs: *n*_FCM_ = 2/40	No	NA	NA
Kim et al. (2023)^54^	HSRs: *n*_FCM_ = 0/43	HSRs: *n*_FCM_ = 0/43	HSRs: *n*_FCM_ = 0/43	No	NA	NA
Talboom et al. (2023)^55^	HSRs: *n*_FCM_ = 0/96	HSRs: *n*_FCM_ = 0/96	HSRs: *n*_FCM_ = 0/96	No	NA	CTCAE (version 5)
Fung et al. (2022)^58^	HSRs: *n*_FDM_ = 0/20	HSRs: *n*_FDM_ = 0/20	HSRs: *n*_FDM_ = 0/20	No	NA	NA
D'Amato et al. (2020)^56^	HSRs: *n*_FCM_ = 0/83	HSRs: *n*_FCM_ = 0/83	HSRs: *n*_FCM_ = 0/83	No	NA	No
Calleja et al. (2016)^57^	HSRs: *n*_FCM_ = 0/111	HSRs: *n*_FCM_ = 0/111	HSRs: *n*_FCM_ = 0/111	No	NA	NA

Abbreviations: AE, adverse event; CTCAE, Common Terminology Criteria for Adverse Events; EudraCT, European Union Drug Regulating Authorities Clinical Trials Database; FCM, ferric carboxymaltose; FDM, ferric (der‐)isomaltose; HP, hypophosphatemia; HSR(s), hypersensitivity reaction(s); MedDRA, Medical Dictionary for Regulatory Activities; NA, not applicable.

^a^ According to Table S4.

^b^ If applicable: Information (e.g., EudraCT protocol or clinical study reports) in addition to publication.

The remaining nine studies did not report any HSRs. However, three studies reported general symptoms without attributing them to HSRs.^49^, ^50^, ^53^ Table 3 highlights where additional sources outside the primary publications were utilized. Figure S1 illustrates the discrepancies among reported symptoms, those potentially associated with HSRs, and those with no association to HSRs.

### 3.6 Reporting

Among the included studies, seven^48^, ^51^, ^52^, ^54^, ^56^, ^57^, ^58^ classified HSRs as AEs, while three^50^, ^52^, ^56^ studies designated anaphylactic reactions as AEs. HP was considered an AE in four studies. One study^52^ categorized HSRs, HP, and anaphylactic reactions collectively as AEs, whereas three studies did not label any of these as AEs.

None of the included studies explicitly mentioned the occurrence or incidence of HSRs, HP, or anaphylactic reactions as predefined outcomes in their methods sections. Most harm‐related outcomes and AEs described in the methods sections were broadly focused on surgical complications, the need for red blood cell transfusions, or general side effects and treatment tolerability.

Two studies^51^, ^54^ applied limitations to AE reporting, either by setting a threshold for reporting or by including only severe events. One study^51^ reported only AEs occurring at a frequency exceeding 5%, a detail not mentioned in the publication but identified in an accompanying EudraCT protocol. For one^56^ cohort study, no information was available regarding potential limitations in AE reporting.

Of the 11 included studies, only three used an established taxonomy for reporting AEs: MedDRA (used by Breymann et al.^48^ and Hansen et al.^51^) and CTCAE (used by Talboom et al.^55^). Two studies^52^, ^57^ restricted AE reporting to the intervention group only. All other studies^48^, ^49^, ^50^, ^51^, ^53^, ^54^, ^55^, ^56^, ^58^ documented and reported AEs, if they occurred, consistently for both intervention and control groups, ensuring comparability.

### 3.7 Summary of reporting practices

Table 4 summarizes key aspects of AE reporting across studies, with a focus on general AE documentation practices. Limitations, such as selective reporting or inconsistent outcome definitions, are highlighted.

**TABLE 4:** Quality assessment of collection and reporting of adverse events.

Author (year)	Explicit designation of specific AEs	Harms and AEs specified as outcomes in methods	Limited reporting^a^	Established outcome taxonomy used?	AEs reporting restriction (e.g., to one study group)	Collection of AEs
Breymann et al. (2016)^48^	HP: yes HSRs: yes Anaphylactic reaction: no^b^/possible yes	Incidence of treatment emerged or related AEs	No	MedDRA (version 16.1)	No	Similar in intervention and control group
Hansen et al. (2022)^51^	HP: yes HSRs: yes Anaphylactic reaction: no^c^/propable yes	Proportion of required RBC transfusion	5% threshold for non‐serious events (only mentioned on EudraCT)	MedDRA (version 20.0)	No	Similar in intervention and control group
Chawla et al. (2022)^49^	HP: no HSRs: no Anaphylactic reaction: no	Adverse effects (intervention and control group)	No	No	No	Similar in intervention and control group
Khalafallah et al. (2018)^50^	HP: yes HSRs: no Anaphylactic reaction: yes	Transfusion requirements tolerability and adverse effects of treatment	No	No	No	Similar in intervention and control group
Thin et al. (2021)^52^	HP: yes HSRs: yes Anaphylactic reaction: yes	Blood transfusion, 30‐day complications and mortality, AEs related to study intervention	No	NA	Intervention group only	Intervention group only
Froessler et al. (2016)^53^	HP: no HSRs: no Anaphylactic reaction: no	Incidence of allogenic blood transfusion, perioperative morbidity, 30‐day mortality, ICU admission	No	NA	No	Not specified
Kim et al. (2023)^54^	HP: no HSRs: yes Anaphylactic reaction: no	Total mediastinal blood loss, transfusion counts, postoperative morbidity and mortality, need for surgical revision	Only serious AEs	NA	No	Not specified
Talboom et al. (2023)^55^	HP: no HSRs: no Anaphylactic reaction: no	Morbidity, reintervention rate, number of transfusions needed, ICU admission	No	CTCAE (version 5)	No	Yes: serious and non‐serious events were collected
Fung et al. (2022)^58^	HP: no HSRs: yes Anaphylactic reaction: no	Number of transfusions Surgical complications Postoperative mortality	No	NA	Not applicable – no AEs occurred	Not specified
D'Amato et al. (2020)^56^	HP: no HSRs: yes Anaphylactic reaction: yes	None	NA	No	Not applicable—no AEs occurred	Not specified
Calleja et al. (2016)^57^	HP: no HSRs: yes Anaphylactic reaction: no	Number of allogenic RBC transfusion, incidence of postoperative complications	No	NA	Yes (to FCM group, which was prospectively surveyed compared to non‐interventional control group, which consisted of retrospectively collected patients)	Not specified

Abbreviations: AE, adverse event; EudraCT, European Union Drug Regulating Authorities Clinical Trials Database; HP, hypophosphatemia; HSR, hypersensitivity reaction; ICU, intensive care unit; NA, not applicable; RBC, red blood cell.

^a^ For example, threshold for reporting, only serious AEs.

^b^ Bronchospasm was not directly stated as an anaphylactic reaction, which is the aim of this item: but should be labeled as “possible” anaphylaxis.

^c^ 2× bronchospasm and flushing arising within the first few minutes of treatment: was not directly labeled as anaphylaxis by the study authors but should be labeled as probable anaphylaxis.

### 3.8 Quality assessment

The quality assessment conducted using the Critical Appraisal Skills Programme (CASP) identified methodological shortcomings in both cohort studies included in the review. Of the two, Calleja et al.^57^ demonstrated greater statistical precision compared to D'Amato et al^56^

For the RCTs, despite some methodological limitations, most studies were of high quality. A common weakness across the included RCTs was inadequate blinding, with inconsistencies in the blinding of participants, investigators, and outcome assessors.

Four studies (Breymann et al., Hansen et al., Khalafallah et al., and Talboom et al.) were notable for their strong methodological rigor. These studies were distinguished by a clearly defined research question, appropriate randomization techniques, and comprehensive outcome reporting.^48^, ^50^, ^51^, ^55^

In contrast, the study by Chawla et al.^49^ exhibited more significant limitations. Key issues included problems with randomization processes and incomplete blinding, which resulted in a lower quality rating compared to the other RCTs.

### 3.9 Summary of quality appraisal

Tables S5 (for RCTs) and S6 (for cohort studies) provide a detailed critical appraisal of the included studies, summarizing their strengths and weaknesses based on CASP criteria.

## 4 DISCUSSION

To the best of our knowledge, this is the first systematic review to focus on the detection of AEs such as HP, HSRs, and anaphylactic reactions following the administration of third‐generation IVI formulations, FDM, and FCM, where laboratory confirmation of ID is required prior to administration.

From the 11 included studies, no conclusions could be drawn regarding the occurrence of HP following the administration of FCM and FDM. This absence of data is attributed to the subclinical nature of HP, coupled with the lack of reported phosphate levels in most trials. Only one study noted 10 cases of low phosphate levels in the FCM treatment group, which were deemed clinically irrelevant.^48^

A total of three potential anaphylactic reactions were reported among 895 patients treated with FCM or FDM. One possible case of anaphylaxis occurred in 775 patients receiving FCM, and two likely cases occurred in 120 patients receiving FDM. Since these three events were not clearly stated as anaphylactic reactions by the study authors, it might be tough for non‐allergologists to identify those events as anaphylactic reactions afterwards. Therefore, a standardized approach with a clear definition of and classification of adverse events is necessary for authors reporting study designs as well as researchers trying to interpret incident cases regarding adverse events.

Recent studies with larger datasets, such as those by Pandey et al. and Kennedy et al., do not contradict our findings. The primary reason for the higher number of included studies in their reviews is that they did not require laboratory confirmation of ID, unlike our review.^21^, ^39^ Pandey et al. concluded that IVI formulations were superior to oral iron supplementation regarding the spectrum of AEs, but did not disaggregate the AEs into specific categories, labeling them only as serious or non‐serious. Furthermore, they did not specifically address the AEs of HP, HSRs, or anaphylactic reactions. Their review also included studies using IVI formulations other than FCM and FDM, which explains the inclusion of 23 additional studies.

Kennedy et al. directly compared the incidence of HSRs following the administration of FDM and FCM. Their analysis of 17 studies indicated a significantly lower incidence of HSRs with FDM compared to FCM, but they concluded that additional head‐to‐head studies were necessary to confirm these findings.^21^ They also noted the lack of data on the frequency and severity of HP following IVI treatment.

These studies highlight the need for research that not only examines the benefits of IVI treatments but also ensures systematic and uniform reporting of AEs. Understanding the mechanisms behind these AEs, such as CARPA, will provide valuable insights for future research and could inform intervention studies in general. Inconsistent data collection and reporting of AEs complicate meta‐analyses, which are already challenged by trial heterogeneity. For HP, the absence of a specific cutoff value at which HP becomes symptomatic further complicates detection. Additionally, some symptoms of HP overlap with those of ID, which can make it difficult to differentiate between the two, especially since HP often presents subclinically.

Despite the challenges associated with capturing and consistently reporting adverse events (AEs), the administration of intravenous iron (IVI) appears to be generally safe. Across more than 700 administrations, only a few severe events (three cases) were documented. While establishing a definitive clinical cutoff for hypersensitivity reactions (HSRs) remains difficult due to their continuous and often overlapping nature, the reported events were predominantly mild and non‐severe. Importantly, only three anaphylactic reactions were observed, and the overall incidence of clinically relevant HSRs was very low.

Nonetheless, several broader safety considerations merit attention in the context of IVI therapy that are not specifically addressed in this systematic review.

1. Hypophosphatemia (HP): Although rarely documented as an AE in the available studies, HP is an emerging concern with certain formulations and in specific settings. It is often subclinical but may become clinically relevant in cases of repeated dosing or prolonged therapy, potentially leading to fatigue, muscle weakness, or osteomalacia.
2. Hypersensitivity and Complement Activation–Related Pseudo‐Allergy (CARPA): True IgE‐mediated reactions are uncommon, but infusion‐related events such as flushing, pruritus, or bronchospasm can occur. Distinguishing mild infusion reactions from severe hypersensitivity or anaphylaxis remains a clinical challenge. At least preparedness for acute management, particularly in pregnant patients or those with multiple comorbidities, seems to be advisable.

The main strength of our Systematic Review is the thorough search of published studies, clinical study reports from the website of regulatory bodies (e.g., European Medicine Agency (EMA)), and entries of clinical trial databases. This approach allows a comprehensive assessment of the incidence of AEs. Furthermore, only studies with laboratory‐confirmed ID were eligible. This ensures that only studies investigating patients with anemia of iron deficiency were included and received appropriate intervention regarding the cause of anemia. The initially planned assessment of reported AEs was adapted due to non‐standardized reporting of AEs in studies. This adapted approach allowed a closer comparison on the collection as well as the reporting (e.g., restriction to a study group or a threshold) of events between studies. Nonetheless, a standardized tool for the evaluation of these important items is currently missing. A limitation of this systematic review, rooted in its strength, is the low number of included studies due to the strict requirement of laboratory confirmation of ID, which led to the exclusion of 73 studies at full‐text screening. The decision to focus only on FCM and FDM also may have contributed to the lack of findings, given the limited scope of included data. The review also highlights the importance of collaboration among researchers, as attempts to contact investigators for additional data, such as clinical study reports, were unsuccessful.

For more valid findings in future research regarding the incidence of AEs, it is crucial to establish standardized terminology and uniform reporting practices for AEs. Facilitating information exchange among researchers will also be important for advancing the quality and depth of evidence in this area.

Considering these findings, it can be concluded that the risks associated with IVI are likely outweighed by the therapeutic benefits, particularly for the treatment of iron deficiency anemia in both pregnant and surgical populations. Future research should emphasize standardized definitions and consistent AE reporting to improve comparability across studies and ensure more precise risk–benefit assessments.

## AUTHOR CONTRIBUTIONS

Dominik Heger: Study selection, data extraction, quality assessment, concept of symptom classification, manuscript drafting, and manuscript revision. Johannes Volkmann: Study selection, data extraction, quality assessment, and manuscript revision. Stephanie Weibel: Concept and study design, search strategy, and manuscript revision. Johanna Stoevesandt: Allergology consultation and manuscript revision. Veronika Walzer: Study selection, data extraction, quality assessment, and manuscript revision. Peter Kranke: Concept and study design, search strategy, and manuscript revision. Patrick Meybohm: Concept and study design, search strategy, and manuscript revision. Stephanie Stangl: Concept and study design, study selection, data extraction, quality assessment, and manuscript revision. Large language models were utilized to enhance the clarity and support the communication of the text.

## FUNDING INFORMATION

This research received no specific grant from any funding agency in the public, commercial, or not‐for‐profit sectors.

## CONFLICT OF INTEREST STATEMENT

Peter Kranke received consulting/speaker's honoraria from Vifor and Pharmacosmos. Patrick Meybohm received consulting/speaker's honoraria from Vifor and Pharmacosmos. Dominik Heger, Stephanie Weibel, Johannes Volkmann, Johanna Stoevesandt, Veronika Walzer, and Stephanie Stangl declare no conflicts of interest.

## Supporting information


**Table S1.** Search strategy—MEDLINE, CENTRAL, clinicaltrials.gov, ICTRP and EUCTR.
**Table S2.** Extracted variables.
**Table S3.** Study characteristics of ongoing studies.
**Table S4.** Symptom‐complex of hypersensitivity reactions.
**Table S5.** CASP—quality assessment for RCTs.
**Table S6.** CASP—quality assessment for cohort studies.
**File S1.** Search strategy—MEDLINE, CENTRAL, clinicaltrials.gov, WHO International Clinical Trials Registry Platform (ICTRP) and EU Clinical Trials Register (EUCTR).

## Data Availability

The data that support the findings of this study are available from the corresponding author upon reasonable request.
