# Supplementary material for: Incidence of adverse events in iron‐deficient pregnant women and surgical patients undergoing intravenous iron treatment with ferric isomaltose or ferric carboxymaltose: A systematic review
Source: Transfusion. 2026 Feb 2;66(4):840–52. doi: 10.1111/trf.70105 (PMC13049238; doi:10.1111/trf.70105)
Supplement: Supplementary file 1 — Table S1. Search strategy—MEDLINE, CENTRAL, clinicaltrials.gov, ICTRP and EUCTR. Table S2. Extracted variables. Table S3. Study characteristics of ongoing studies. Table S4. Symptom‐complex of hypersensitivity reactions. Table S5. CASP—quality assessment for RCTs. Table S6. CASP—quality assessment for cohort studies. File S1. Search strategy—MEDLINE, CENTRAL, clinicaltrials.gov, WHO International Clinical Trials Registry Platform (ICTRP) and EU Clinical Trials Register (EUCTR). [file TRF-66-840-s001.docx]

**Incidence of Adverse Events in Iron-Deficient Pregnant Women and Surgical Patients Undergoing Intravenous Iron Treatment with Ferric Isomaltose or Ferric Carboxymaltose: A Systematic Review**

Supplemental Table 1. Search strategy – MEDLINE, CENTRAL, clinicaltrials.gov, ICTRP and EUCTR.

Supplemental Table 2. Extracted variables.

Supplemental Table 3. Study characteristics of ongoing studies.

Supplemental Table 4. Symptom-complex of hypersensitivity reactions.

Supplemental Table 5. CASP- quality assessment for RCTs.

Supplemental Table 6. CASP- quality assessment for cohort studies.

**Supplementary file 1. Search strategy – MEDLINE, CENTRAL, clinicaltrials.gov, WHO International Clinical Trials Registry Platform (ICTRP) and EU Clinical Trials Register (EUCTR)**

Ovid MEDLINE(R) and Epub Ahead of Print, In-Process, In-Data-Review & Other Non-Indexed Citations, Daily and Versions <1946 to May 24, 2023>

Search Date: 05/25/2023

Supplemental Table 1: Search strategy

| Search | Query | Results |
| --- | --- | --- |
| 1 | (preoperat* or pre operat* or pre-operat* or preprocedur* or pre clinical* or pre-clinical* or preclinical* or pre-surg* or presurg* or surgical or surgery or operat*).mp. | 4487684 |
| 2 | Preoperative Care/ or Preoperative Period/ or exp Perioperative Period/ or exp Specialties, Surgical/ | 380228 |
| 3 | 1 or 2 | 4588321 |
| 4 | (matern* or gestation* or prenatal* or pre-natal* or obstetric* or pre-part* or prepart* or antenatal* or pregnan*).mp. | 1400843 |
| 5 | exp Pregnancy/ or exp Pregnancy Complications/ or exp Maternal Health Services/ or Perinatal Care/ | 1044042 |
| 6 | 4 or 5 | 1436328 |
| 7 | 3 or 6 | 5864033 |
| 8 | Iron Deficiencies/bl, di, dt, th [Blood, Diagnosis, Drug Therapy, Therapy] | 38 |
| 9 | Iron/ or *Iron Deficiencies/ or Anemia/di, th [Diagnosis, Therapy] | 114373 |
| 10 | (iron depletion or iron deficien*).ti,ab,kf. | 26519 |
| 11 | Anemia, Iron-Deficiency/pc, di, dt | 5873 |
| 12 | 8 or 9 or 10 or 11 | 129574 |
| 13 | 7 and 12 | 15786 |
| 14 | exp Iron Compounds/ | 70659 |
| 15 | (iron or ferric or ferrous or Monofer or Ferinject or isomaltoside or derisomaltose or carboxymaltose or iron supplementation or iron treatment).ab,ti,kf. | 243042 |
| 16 | 14 or 15 | 285560 |
| 17 | 13 and 16 | 12033 |
| 18 | 17 not (Animals/ not (Animals/ and Humans/)) | 10747 |
| 19 | Epidemiologic studies/ | 9326 |
| 20 | exp cohort studies/ | 2482641 |
| 21 | (cohort adj (study or studies)).tw. | 311797 |
| 22 | Cohort analy$.tw. | 11662 |
| 23 | (Follow up adj (study or studies)).tw. | 56007 |
| 24 | (observational adj (study or studies)).tw. | 158957 |
| 25 | Longitudinal.tw. | 319257 |
| 26 | Retrospective.tw. | 736023 |
| 27 | or/19-26 | 3006248 |
| 28 | Randomized Controlled Trials as Topic/ | 162082 |
| 29 | randomized controlled trial/ | 593127 |
| 30 | Random Allocation/ | 106929 |
| 31 | Double Blind Method/ | 175218 |
| 32 | Single Blind Method/ | 32706 |
| 33 | clinical trial/ | 538010 |
| 34 | clinical trial, phase i.pt. | 24880 |
| 35 | clinical trial, phase ii.pt. | 39676 |
| 36 | clinical trial, phase iii.pt. | 21702 |
| 37 | clinical trial, phase iv.pt. | 2414 |
| 38 | controlled clinical trial.pt. | 95308 |
| 39 | randomized controlled trial.pt. | 593127 |
| 40 | multicenter study.pt. | 333950 |
| 41 | clinical trial.pt. | 538010 |
| 42 | exp Clinical Trials as topic/ | 382243 |
| 43 | or/28-42 | 1562831 |
| 44 | (clinical adj trial$).tw. | 474184 |
| 45 | ((singl$ or doubl$ or treb$ or tripl$) adj (blind$3 or mask$3)).tw. | 196831 |
| 46 | PLACEBOS/ | 35928 |
| 47 | placebo$.tw. | 246023 |
| 48 | randomly allocated.tw. | 36113 |
| 49 | (allocated adj2 random$).tw. | 39862 |
| 50 | or/44-49 | 777196 |
| 51 | 43 or 50 | 1905527 |
| 52 | case report.tw. | 393810 |
| 53 | letter/ | 1217493 |
| 54 | historical article/ | 369284 |
| 55 | 52 or 53 or 54 | 1961693 |
| 56 | 51 not 55 | 1863026 |
| 57 | 27 or 56 | 4417764 |
| 58 | 18 and 57 | 2924 |
| 59 | limit 58 to yr="2000 -Current" | 2432 |
| 60 | limit 59 to (english or german) | **2332** |

Cochrane Centre Register of Controlled Trials (CENTRAL) (via Cochrane Library):

Search Name: IV_iron:AE_25052023_1
Search Date: 05/25/2023

| ID | | Search | | Results |
| --- | --- | --- | --- | --- |
| #1 | MeSH descriptor: [Preoperative Care] this term only | | 5016 | |
| #2 | MeSH descriptor: [Perioperative Period] explode all trees | | 12239 | |
| #3 | MeSH descriptor: [Preoperative Period] this term only | | 763 | |
| #4 | MeSH descriptor: [Specialties, Surgical] explode all trees | | 3687 | |
| #5 | (preoperat* OR pre operat* OR pre-operat* OR preprocedur* OR pre clinical* OR pre-clinical* OR preclinical* OR pre-surg* OR presurg* OR surgical OR surgery OR operat*):ti,ab,kw | | 408781 | |
| #6 | (matern* OR gestation* OR prenatal* or pre-natal* OR obstetric* or pre-part* OR prepart* or antenatal* OR pregnan*):ti,ab,kw | | 106284 | |
| #7 | MeSH descriptor: [Pregnancy] explode all trees | | 31028 | |
| #8 | MeSH descriptor: [Pregnancy Complications] explode all trees | | 15881 | |
| #9 | MeSH descriptor: [Maternal Health Services] explode all trees | | 3214 | |
| #10 | MeSH descriptor: [Perinatal Care] this term only | | 309 | |
| #11 | #1 or 2# or #3 or #4 or #5 | | 1334447 | |
| #12 | #6 or #7 or #8 or #9 or #10 or #11 | | 1364953 | |
| #13 | #11 or #12 | | 1364953 | |
| #14 | MeSH descriptor: [Iron Deficiencies] explode all trees and with qualifier(s): [blood - BL, diagnosis - DI, drug therapy - DT, therapy - TH] | | 780 | |
| #15 | MeSH descriptor: [Iron] explode all trees | | 2936 | |
| #16 | MeSH descriptor: [Iron Deficiencies] explode all trees | | 1842 | |
| #17 | MeSH descriptor: [Anemia] this term only and with qualifier(s): [therapy - TH, diagnosis - DI] | | 403 | |
| #18 | (iron depletion OR iron deficien*):ti,ab,kw | | 5173 | |
| #19 | #14 or #15 or #16 or #17 or #18 | | 6933 | |
| #20 | #13 and #19 | | 5161 | |
| #21 | MeSH descriptor: [Iron Compounds] explode all trees | | 2685 | |
| #22 | (iron OR ferric OR ferro OR Monofer OR Ferinject OR isomaltoside OR derisomaltose OR carboxymaltose OR iron supplementation OR iron treatment):ti,ab,kw | | 12515 | |
| #23 | #21 or #22 | | 13506 | |
| #24 | #20 and #23 with Publication Year from 2000 to present, in Trials | | **4123** | |

clinicaltrials.gov:

**120** Studies found for:
**Completed Studies | Iron Deficiency Anemia of Pregnancy** OR **iron deficiency of Pregnancy** OR **anemia of Pregnancy** OR **iron deficiency** OR **iron deficiency anemia** OR **anemia | carboxymaltose** OR **isomaltoside** OR **derisomaltose** OR **iron** OR **ferro** OR **ferric | Adult, Older Adult |** Results first posted from 01/01/2000 to 05/25/2023

ICTRP:

**115** records for **86** trials found for:
**isomaltoside**; isomaltoside || **ferric**; ferric || **ferro**; ferro ||**derisomaltose**; derisomaltose || **carboxymaltose**; carboxymaltose
Date Range: from 01/01/2000 to 05/25/2023

EUCTR:

**70** result(s) found for:
**iron defieciency** OR **iron deficiency anemia** OR **anemia** OR **anaemia** OR **carboxymaltose** OR **isomaltoside** OR **derisomaltoside** OR **anemia of pregnancy** OR **iron deficiency of pregnancy** OR **iron** OR **ferro** OR **ferric**Date Range: from 01/01/2000 to 05/25/2023

Supplemental Table 2: Extracted variables

| Category | Sub-Category | Variables |
| --- | --- | --- |
| Study characteristics | Extraction | 1^st^ extractor, 2^nd^ extractor \| 3^rd^ extractor; date of extraction and date of check |
|  | Study-ID | Covidence-number and Trial name (if applicable) |
|  | Study information | Author; year; Title; Registry1; Registry2; Authors (full list); Source (Journal); Publication type; DOI or URL; comments |
|  | Study design and details | Study type – RCT/cohort; Trial Phase; country/countries (if multicentral); number of all patients included; number of IDA/ID participants receiving intervention/comparator (n (%)); number of analyzed IDA/ID participants (n); funding; conflict of interests of authors |
|  | PICO summary | Population; Intervention; Comparator; Primary outcome (as defined in the methods section); Secondary outcomes (as defined in the methods section) |
|  | Population characteristics | Inclusion criteria; Exclusion criteria; Definition of anemia; Diagnostic criteria for ID; Age (mean±SD); Age (median & range (IQR or min-max)); trimenon in pregnancy, parity, singleton or multiple \| Type of surgery; comments |
|  | Intervention description | Intervention; Dose; Frequency; Route of administration; Comments |
|  | Comparator description | Comparator; Describe usual/standard care; comments |
| Outcome | Study-ID | Covidence-number, Author; Study / Trial name; |
| Outcome | Outcome characteristics | Outcome type (PO, SO); Definition of outcome; Outcome specified in method section; Outcome definition according to registry (if registered); Outcome taxonomy; Outcome assessed by (e.g., person); Timepoint/(s) outcome measures / duration; instruments / kits /standardized questionnaire / medical records for measuring outcome; Outcome measure (reported); Source; Direction of result (significant benefit= sig-benefit, significant harm = sig-harm, non-significant = ns, not reported), if more than one measure reported indicate how many significant; comments |
| Outcome | AEs | Incidence; severity; need for treatment; need for drop out; phosphate-levels in case of HP; symptoms for description of HSRs or anaphylactic reactions if reported;comments |
| Abbreviations: RCT: randomized controlled trial; ID(A): iron deficiency (anemia); n: number (e.g., of patients); SD: standard deviation; IQR: interquartile range; PO: primary outcome; SO: secondary outcome; AEs: adverse events; HP: hypophosphatemia; HSRs: hypersensitivity reactions | | |

Supplemental Table 3: Ongoing studies

| Titel | Primary sponsor | Registry number | Drug | Reference | Estimated completion |
| --- | --- | --- | --- | --- | --- |
| Reducing Anemia in Pregnancy in India: the RAPIDIRON Trial | Thomas Jefferson University | NCT05358509 | FCM/FDM | Derman, et al. ^1^ | 31.06.2024 |
| Reducing Anemia in Pregnancy in India: the RAPIDIRON Trial | Childrens Investment Fund Foundation | CTRI/2020/09/027730 | FCM/FDM | Bellad ^2^ | 31.01.2024 |
| Intraveous Versus Oral Iron for Treating Iron-Deficiency Anemia in Pregnancy (IVIDA2) | Women and Infants Hospital of Rhode Island | NCT05462704 | FDM | II: Lewkowitz, et al. ^3,4^ | 30.04.2027 |
| The IRONWOMAN pilot feasibility study: oral versus intravenous iron therapy for iron deficiency anaemia in late pregnancy | The National Blood Authority, Australia | ACTRN12619000283178 | FCM | ^5^ | N/A  -- Waiting for Approval |
| Ferritin Screening And IRon Treatment for Maternal Anemia and Fetal Growth Restriction (FGR) Prevention - a Multi Centre Randomised Controlled Trial (RCT) | Services Institute of Medical Sciences, Pakistan | NCT04228627 | NA | II: Record and Preprint ^6^ | 28.02.2024 |
| Iron infusion in iron-deficient patients undergoing surgery for colorectal cancer who are anaemic versus not anaemic in order to improve physical fitness as determined by exercise testing and a quality of life questionnaire (FeRIC) | Peter MacCallum Cancer Centre Foundation Grant | ACTRN12616001219471 | FCM | ^7^ | Anticipated 20/07/2020 |
| Intravenous iron for treatment of Sub-optimal iron stores in Non-Anaemic Patients presenting for major Surgery (I-SNAPS) | National Blood Authority Australia, The University of Western Australia | ACTRN12620000314921 | FCM | ^8^ | Not yet recruiting |
| Efficacy of preoperative intravenous iron isomaltoside in colorectal cancer surgical patients with iron deficiency anaemia compared to standard care: a pilot randomized controlled trial | Department of Anaesthesia and Intensive Care, 4/F, Prince of Wales Hospital, Shatin, N.T. Hong Kong, China | ChiCTR1800017010 | FDM | ^9^ | Anticipated 30/04/2021 |
| Treatment of anemia (low red blood cells) with iron infusions in patients listed for a liver transplantation | Medical University of Vienna | EUCTR2017-000703-24-AT | FCM | ^10^ | Prematurely Ended |
| Effect of intravenous replenishment of iron in preoperative management of anemia in patients with colon cancer: rIPAC-trial | Vifor Pharma  University Hospital Ghent | EUCTR2018-004213-41-BE | FCM | ^11^ | Final study report available |
| A study of intravenous iron isomaltoside 1000 (Monofer®) compared to placebo in subjects with iron deficiency anaemia who are Intolerant or unresponsive to oral iron therapy | Pharmacosmos A/S | EUCTR2014-001518-25-DE | FDM | ^12^ | Prematurely Ended |
| A Placebo Study Comparing Intravenous Iron with Saline in Treatment of Low Blood Count before Surgery in Patients with Cancer of the Kidney, Bladder or Lower Abdominal Cavity | Pharmacosmos A/S | EUCTR2013-004979-13-DK | FDM | ^13^ | Prematurely Ended |
| Effects of intravenous administered iron in non-anemic iron deficient patients with colorectal cancer | Centre for Surgical Science, Pharmacosmos A/S | EUCTR2019-003819-56-DK | FDM | ^14^ | NA |
| Iron Deficiency in cardiac surgery | UNIVERSITÀ CAMPUS BIO-MEDICO DI ROMA | EUCTR2017-001823-26-IT | FCM | ^15^ | Prematurely Ended |
| Multicenter randomized controlled trial comparing intravenous iron infusion with oral iron suppletion in the preoperative work-up of patients with anemia that undergo surgery for cancer in the bowel | Academic Medical Center, Vifor Pharma | EUCTR2014-002827-87-NL | FCM | ^16^ | NA |
| Impact of Intravenous Iron Treatment of Preoperative Anemia in Patients With LEAD (IRONPAD) | Biocruces Bizkaia Health Research Institute | NCT04083755 | FCM | ^17^ | NA |
| Preoperative Intravenous Iron Therapy in Patients With Gastric Cancer | Helsinki University Central Hospital | NCT04168346 | FCM | ^18^ | Anticipated 11/2026 |
| Effect of Preoperative Intravenous Ferric Carboxymaltose for Clipping Surgery (PICASA) | Seoul National University Hospital, Vifor Pharma | NCT04616092 | FCM | ^19^ | Anticipated 04/2023 |
| Preoperative i.v. Iron Substitution in Patients With Colon Cancer (PREFECO) | Helsinki University Central Hospital | NCT04653181 | FCM | ^20^ | Anticipated 31/12/2025 |
| Ferric Derisomaltose and Outcomes in the Recovery of Gynecologic Oncology: ERAS (Enhanced Recovery After Surgery) | AHS Cancer Control Alberta | NCT05407987 | FCM | ^21^ | Anticipated 30/12/2025 |
| Ferric Derisomaltose/Iron Isomaltoside and Outcomes in the Recovery of Gynecologic Oncology ERAS (FORGE) | Alberta Health Services, Calgary | NCT05467319 | FDM | ^22^ | Anticipated 12/2023 |
| Effects of Intravenous Administered Iron in Non-anemic Iron Deficient Patients With Colorectal Cancer (NAIDIC) | Zealand University Hospital | NCT05220800 | FDM | ^23^ | Anticipated 01/03/2029 |
| Effect of preoperative intravenous administration of iron in patients with mild anemia undergoing arterial vascular surgery | VU Medical Center | NTR4185 | FCM | ^24^ | NA |
| **Abbreviations**: FCM: ferric carboxymaltose (Ferinject®); FDM: ferric derisomaltose (MonoFer®) | | | | | |

Supplemental Table 4: Complex of HSRs-Spectrum

| **Symptom spectrum of anaphylaxis according to guidelines/literature^25-30^** | | | | |
| --- | --- | --- | --- | --- |
| **Cutaneous/mucocutaneous** | **Respiratory** | **Cardiovascular** | **Gastrointestinal** | **Unspecific** |
| Urticaria  Angioedema  Flush  Pruritus  Conjunctival injection | Dyspnea  Wheeze/bronchospasm  Stridor/laryngospasm  Hypoxemia | Hypotonia (blood pressure < 90 mmHg)  Tachycardia  Syncope/loss of consciousness (possibly associated with urine incontinence) | Crampy abdominal pain  Diarrhea  Vomiting | Anxiety  Perception of impending doom  Perception of “pins and needles”/burning  Metallic taste  Confusion |
| Anaphylaxis was considered “likely” in the case of acute onset (during or within the first hour after intravenous iron treatment) of at least 2 specific symptoms. It was considered “possible” in the case of acute onset of 1 specific symptom. | | | | |

| **Symptom spectrum of HSRs according to guidelines/literature^31,32^** | |
| --- | --- |
| **Early onset** | **Late onset** |
| Signs and symptoms of anaphylaxis | Maculopapular exanthem  Acute generalized exanthematous pustulosis  Drug reaction with eosinophilia and systemic symptoms  Fixed drug eruption  Stevens-Johnson syndrome / Toxic epidermal necrolysis |
| **Abbreviations**: HSR(s): hypersensitivity reaction(s); | |

Supplemental Table 5: CASP- quality-assessment for RCTs

| CASP checklist for qualitative research summary table ––– randomized controlled studies | | | | | | | | | |
| --- | --- | --- | --- | --- | --- | --- | --- | --- | --- |
| ITEM | **Breymann^33^  ––  2017** | **Chawla ^34^  ––  2022** | **Hansen ^35^  ––  2023** | **Khalafallah^36^  ––  2018** | **Thin ^37^  –– 2021** | **Froessler^38^ –– 2016** | **Kim ^39^  –– 2023** | **Talboom ^40^ –– 2023** | **Fung ^41^  –– 2022** |
| Did the study address a clearly focused research question? | **YES** | **NO** | **YES** | **YES** | **YES** | **YES** | **YES** | **YES** | **YES** |
| Was the assignment of participants to interventions randomised? | **YES** | **NA** | **YES** | **YES** | **YES** | **YES** | **YES** | **YES** | **YES** |
| Were all participants who entered the study accounted for at its conclusion? | **YES** | **NO** | **YES** | **YES** | **YES** | **YES** | **YES** | **YES** | **YES** |
| Were the participants ‘blind’ to intervention they were given? | **NO** | **NA** | **NO** | **NO** | **NO** | **NO** | **YES** | **NA** | **NO** |
| Were the investigators ‘blind’ to the intervention they were giving to participants? | **NO** | **NA** | **NO** | **NO** | **NO** | **NA** | **NA** | **NA** | **NA** |
| Were the people assessing/analysing outcome/s ‘blinded”? | **NA** | **NA** | **NA** | **YES** | **NO** | **YES** | **NA** | **NA** | **YES** |
| Were the study groups similar at the start of the randomised controlled trial? | **YES** | **YES** | **YES** | **YES** | **NO** | **YES** | **YES** | **YES** | **YES** |
| Apart from the experimental intervention, did each study group receive the same level of care (that is, were they treated equally)? | **YES** | **NO** | **YES** | **YES** | **YES** | **YES** | **YES** | **YES** | **YES** |
| Were the effects of intervention reported comprehensively? | **YES** | **YES** | **YES** | **YES** | **YES** | **YES** | **YES** | **YES** | **YES** |
| Was the precision of the estimate of the intervention or treatment effect reported? | **YES** | **YES** | **YES** | **YES** | **YES** | **YES** | **YES** | **YES** | **YES** |
| Do the benefits of the experimental intervention outweigh the harms and costs? | **YES** | **YES** | **YES** | **YES** | **NA** | **NA** | **NA** | **YES** | **NA** |
| Can the results be applied to your local population/in your context? | **YES** | **NO** | **YES** | **YES** | **NO** | **NA** | **NA** | **YES** | **NA** |
| Would the experimental intervention provide greater value to the people in your care than any of the existing interventions? | **YES** | **NA** | **YES** | **YES** | **NO** | **NA** | **NA** | **NA** | **NA** |
| Appraisal summary | ***1** | ***2** | ***3** | ***4** | ***5** | ***6** | ***7** | ***8** | ***9** |
| Abbreviations: NA: not applicable; Hb: hemoglobin; FCM: ferric carboxymaltose; CI: confidence intervals  *1: Sound study design with a clear research question, but limitations in blinding and group comparability. Comprehensive reporting of results, but unclear whether benefits outweigh harms and costs. Strong positive outcomes lead to high study quality.  *2: Detailed methodology, but issues with randomization and blinding. Comprehensive outcome reporting, but lacking cost-benefit analysis. Applicability is unclear. Low to moderate study quality due to missing information.  *3: Clear research question with a strong design, though blinding is incomplete. Consistently positive results contribute to high study quality.  *4: Solid design based on a specific research question with predominantly positive evaluations despite partial blinding issues. Very high study quality.  *5: Clear research question and solid foundation, but weaknesses in blinding. Mixed evaluations lead to moderate study quality.  *6: Clear research question with appropriate randomization, but no participant blinding. Comprehensive reporting, though unclear on the benefit-risk ratio. High study quality despite limitations.  *7: Clear research question with strengths in design; complete blinding is a plus. Some aspects remain unassessed. Medium to high quality depending on further information.  *8: Good foundation with a clear research question, but many unassessable aspects regarding blinding. Very positive results lead to medium to high quality.  *9: Clear research question with strengths in randomization and data collection, but partial lack of blinding. Predominantly positive methods and results result in high study quality. | | | | | | | | | |

Supplemental Table 6: CASP- quality-assessment for cohort studies

| CASP checklist for qualitative research summary table ––– cohort studies | | |
| --- | --- | --- |
| ITEM | **D’ Amato ^42^ –– 2020** | **Calleja ^43^ –– 2016** |
| Did the study address a clearly  focused issue? | **YES** | **YES** |
| Was the cohort recruited in  an acceptable way? | **YES** | **NO** |
| Was the exposure accurately  measured to minimize bias? | **NO** | **YES** |
| Was the outcome accurately  measured to minimize bias? | **YES** | **NA** |
| Have the authors identified  all important confounding  factors? | **NO** | **NA** |
| Have they taken account of  the confoundding factors in the  design and/or analysis? | **NO** | **NA** |
| Was the follow up of  subjects complete enough? | **YES** | **YES** |
| Was the follow up of  subjects long enough? | **YES** | **YES** |
| What are the results of this study? | Quicker restoration of [Hb] with FCM | Less transfusion rate due to FCM administration; improved clinical outcome |
| How precise are the results? | No CI are provided | Statistically significant, with 95% CI |
| Do you believe the results? | **YES** | **YES** |
| Can the results be applied to  the local population? | **YES** | **YES** |
| Do the results of this study fit with other available evidence? | **NA** | **YES** |
| What are the implications of this study for practice? | **NA** | **YES** |
| Abbreviations: NA: not applicable; Hb: hemoglobin; FCM: ferric carboxymaltose; CI: confidence intervalls | | |

1. Derman RJ, Goudar SS, Thind S, et al. RAPIDIRON: Reducing Anaemia in Pregnancy in India—a 3-arm, randomized-controlled trial comparing the effectiveness of oral iron with single-dose intravenous iron in the treatment of iron deficiency anaemia in pregnant women and reducing low birth weight deliveries. *Trials*. 2021;22(1):649.

2. Bellad DMB. Reducing Anemia in Pregnancy in India: the RAPIDIRON Trial 10/09/2020.

3. Lewkowitz AK, Stout MJ, Carter EB, et al. Protocol for a multicenter, double-blinded placebo-controlled randomized controlled trial comparing intravenous ferric derisomaltose to oral ferrous sulfate for the treatment of iron deficiency anemia in pregnancy: The IVIDA2 trial. *Contemporary Clinical Trials*. 2022;123:106992.

4. Methodius Tuuli M, MPH, MBA. Double-blind Placebo-controlled Multicenter Randomized Trial of Intravenous Versus Oral Iron for Treating Iron-Deficiency Anemia in Pregnancy; 2022.

5. Shand DA. The IRONWOMAN pilot feasibility study: a double blind randomised trial to compare feasibility of blinding of intravenous or oral iron replacement to placebo intravenous or oral therapy for iron deficiency anaemia in pregnancy: Main Sponsor: The National Blood Authority, Australia; 2019.

6. Wasim T, Bushra N, Tajammul A, et al. Ferritin screening and Iron treatment for maternal anemia and fetal growth restriction prevention - A multicenter randomized controlled trial (FAIR Study). *Pakistan Journal of Medical Sciences*. 2022;39(1).

7. Bolshinsky V. An iron infusion with Ferric Carboxymaltose as a technique of improving physiological reserve in iron deficient patients undergoing surgery for colorectal cancer: a randomized control trial; 2024.

8. Mace DH. Intravenous iron for treatment of Sub-optimal iron stores in Non-Anaemic Patients presenting for major Surgery; 2024.

9. Polly DFPL. Efficacy of preoperative intravenous iron isomaltoside in colorectal cancer surgical patients with iron deficiency anaemia compared to standard care: a pilot randomized controlled trial; 2024.

10. Vienna MU. Treatment of anemia with intravenous iron in patients listed for

orthotopic liver transplantation; 2024.

11. Geboes PDK. Effect of intravenous replenishment of iron in the preoperative management of anemia in patients with colon cancer: RIPAC-trial 2024.

12. BfArM G-. A Phase III, Randomised, Double-blind, Comparative Study of Intravenous Iron Isomaltoside 1000 (Monofer®) against Placebo in Subjects with Iron Deficiency Anaemia and who are Intolerant or Unresponsive to Oral Iron Therapy; 2024.

13. DHMA D-. Treatment of Preoperative Anaemia in Patients with Urogenital Cancer: A Randomised Double-Blind Placebo-Controlled Study of Intravenous Iron Isomaltide 1000 Monofer© versus Saline; 2024.

14. DHMA D-.

Effects of intravenous administered iron in non-anemic iron deficient patients with colorectal cancer. A double blinded clinical randomized trial.; 2024.

15. Agency I-IM. Efficacy and Safety of Intravenous Ferric Carboxymaltose in Patients

Undergoing Cardiac Surgery A Randomized Controlled Study; 2024.

16. Authority N-C. Multicenter randomized controlled trial comparing Ferric(III)carboxymaltose infusion with oral iron suppletion in the treatment of preoperative anemia in colorectal cancer patients; 2024.

17. AEMPS S-. IMPACT OF INTRAVENOUS IRON TREATMENT OF PREOPERATIVE ANEMIA IN PATIENTS WITH LOWER EXTREMITY PERIPHERAL ARTERY DISEASE; 2024.

18. Arto Kokkola M. Preoperative Intravenous Iron Therapy in Patients With Gastric Cancer; 2024.

19. Si Un Lee P. Effect of Preoperative Intravenous Ferric Carboxymaltose on Postoperative Transfusion Reduction in Anemia Patients Scheduled for Clipping Surgery for Unruptured Intracranial Aneurysms; 2024.

20. Suvi K Rasilainen M, PhD. Preoperative i.v. Iron Substitution in Patients With Colon Cancer (PREFECO); 2024.

21. Steven Bisch M. Ferric Derisomaltose and Outcomes in the Recovery of Gynecologic Oncology: ERAS (Enhanced Recovery After Surgery); 2024.

22. Steven Bisch M. Ferric Derisomaltose/Iron Isomaltoside and Outcomes in the Recovery of Gynecologic Oncology ERAS (FORGE); 2024.

23. Hospital ZU. Effects of Intravenous Administered Iron in Non-anemic Iron Deficient Patients With Colorectal Cancer (NAIDIC); 2024.

24. Boer C. Effect of preoperative intravenous administration of iron in patients with mild anemia undergoing arterial vascular surgery; 2024.

25. Szebeni J, Fishbane S, Hedenus M, et al. Hypersensitivity to intravenous iron: classification, terminology, mechanisms and management. *Br J Pharmacol*. 2015;172(21):5025-5036.

26. Achebe M, DeLoughery TG. Clinical data for intravenous iron - debunking the hype around hypersensitivity. *Transfusion*. 2020;60(6):1154-1159.

27. Trautmann A, Kleine-Tebbe J. Allergologie in Klinik und Praxis: Allergene-Diagnostik-Therapie: Georg Thieme Verlag; 2022.

28. Belmont A, Liao J, Hsu FI, Kwah J. A safe, effective, and single-day protocol for rapid drug desensitization to intravenous iron dextran. *The Journal of Allergy and Clinical Immunology: In Practice*. 2023;11(10):3242-3244.e3241.

29. Ring J, Beyer K, Biedermann T, et al. Leitlinie zu Akuttherapie und Management der Anaphylaxie-Update 2021: S2k-Leitlinie der Deutschen Gesellschaft für Allergologie und klinische Immunologie (DGAKI), des Ärzteverbands Deutscher Allergologen (AeDA), der Gesellschaft für Pädiatrische Allergologie und Umweltmedizin (GPA), der Deutschen Akademie für Allergologie und Umweltmedizin (DAAU), des Berufsverbands der Kinder-und Jugendärzte (BVKJ), der Gesellschaft für Neonatologie und Pädiatrische Intensivmedizin (GNPI), der Deutschen Dermatologischen Gesellschaft (DDG), der Österreichischen Gesellschaft für Allergologie und Immunologie (ÖGAI), der Schweizerischen Gesellschaft für Allergologie und Immunologie (SGAI), der Deutschen Gesellschaft für Anästhesiologie und Intensivmedizin (DGAI), der Deutschen Gesellschaft für Pharmakologie (DGP), der Deutschen Gesellschaft für Pneumologie und Beatmungsmedizin (DGP), der Patientenorganisation Deutscher Allergie-und Asthmabund (DAAB) und der Arbeitsgemeinschaft Anaphylaxie-Training und Edukation (AGATE). *Allergo Journal*. 2021;30:20-49.

30. Muraro A, Worm M, Alviani C, et al. EAACI guidelines: anaphylaxis (2021 update). *Allergy*. 2022;77(2):357-377.

31. Brockow K, Wurpts G, Trautmann A, et al. Guideline for allergological diagnosis of drug hypersensitivity reactions: S2k guideline of the German society for allergology and clinical immunology (DGAKI) in Cooperation with the German dermatological society (DDG), the association of German allergologists (ÄDA), the German society for pediatric allergology (GPA), the German contact dermatitis research group (DKG), the German society for pneumology (DGP), the German society of otorhinolaryngology, head and neck surgery, the Austrian society of allergology and immunology (ÖGAI), the Austrian society of dermatology and venereology (ÖGDV), the German academy of allergology and environmental medicine (DAAU), and the German Documentation center for severe skin reactions (dZh). *Allergologie select*. 2023;7:122.

32. Demoly P, Adkinson NF, Brockow K, et al. International Con sensus on drug allergy. *Allergy*. 2014;69(4):420-437.

33. Breymann C, Milman N, Mezzacasa A, Bernard R, Dudenhausen J. Ferric carboxymaltose vs. oral iron in the treatment of pregnant women with iron deficiency anemia: an international, open-label, randomized controlled trial (FER-ASAP). *J Perinat Med*. 2017;45(4):443-453.

34. Chawla S, Singh A, Jhamb D, Anupama CH. A Randomised Controlled Trial to Compare Injection Ferric Carboxymaltose and Oral Iron in Treating Iron Deficiency Anemia During Pregnancy. *The Journal of Obstetrics and Gynecology of India*. 2022;72(6):492-496.

35. Hansen R, Sommer VM, Pinborg A, et al. Intravenous ferric derisomaltose versus oral iron for persistent iron deficient pregnant women: a randomised controlled trial. *Archives of Gynecology and Obstetrics*. 2023;308(4):1165-1173.

36. Khalafallah AA, Hyppa A, Chuang A, et al. A Prospective Randomised Controlled Trial of a Single Intravenous Infusion of Ferric Carboxymaltose vs Single Intravenous Iron Polymaltose or Daily Oral Ferrous Sulphate in the Treatment of Iron Deficiency Anaemia in Pregnancy. *Seminars in Hematology*. 2018;55(4):223-234.

37. Thin TN, Tan BPY, Sim EY, Shum KL, Chan HSP, Abdullah HR. Preoperative Single-Dose Intravenous Iron Formulation to Reduce Postsurgical Complications in Patients Undergoing Major Abdominal Surgery: A Randomized Control Trial Feasibility Study (PIRCAS Trial Pilot). *Cureus*. 2021;13(8):e17357.

38. Froessler B, Palm P, Weber I, Hodyl NA, Singh R, Murphy EM. The Important Role for Intravenous Iron in Perioperative Patient Blood Management in Major Abdominal Surgery: A Randomized Controlled Trial. *Ann Surg*. 2016;264(1):41-46.

39. Kim HH, Park EH, Lee SH, Yoo KJ, Youn YN. Effect of Preoperative Administration of Intravenous Ferric Carboxymaltose in Patients with Iron Deficiency Anemia after Off-Pump Coronary Artery Bypass Grafting: A Randomized Controlled Trial. *J Clin Med*. 2023;12(5).

40. Talboom K, Borstlap WAA, Roodbeen SX, et al. Ferric carboxymaltose infusion versus oral iron supplementation for preoperative iron deficiency anaemia in patients with colorectal cancer (FIT): a multicentre, open-label, randomised, controlled trial. *Lancet Haematol*. 2023;10(4):e250-e260.

41. Fung PLP, Lau VNM, Ng FF, Leung WW, Mak TWC, Lee A. Perioperative changes in haemoglobin and ferritin concentrations from preoperative intravenous iron isomaltoside for iron deficiency anaemia in patients with colorectal cancer: A pilot randomised controlled trial. *PLoS One*. 2022;17(6):e0270640.

42. D'Amato T, Kon E, Martorelli F, et al. Effect of intravenous ferric carboxymaltose supplementation in non-anaemic iron deficient patients undergoing hip and knee arthroplasty. *J Biol Regul Homeost Agents*. 2020;34(4 Suppl. 3):69-77. Congress of the Italian Orthopaedic Research Society.

43. Calleja JL, Delgado S, del Val A, et al. Ferric carboxymaltose reduces transfusions and hospital stay in patients with colon cancer and anemia. *Int J Colorectal Dis*. 2016;31(3):543-551.
